# Supplementary material for: When spatial attention cannot be divided: Quadrantic enhancement of early visual processing across task-Relevant and irrelevant locations
Source: Imaging Neurosci (Camb). 2024 Jun 20;2:imag-2-00194. doi: 10.1162/imag_a_00194 (PMC12272178; doi:10.1162/imag_a_00194)
Supplement: Supplementary Material [file imag_a_00194-supp.pdf]

Supplemental Materials

HEOG Activity in Experiments 2 and 3

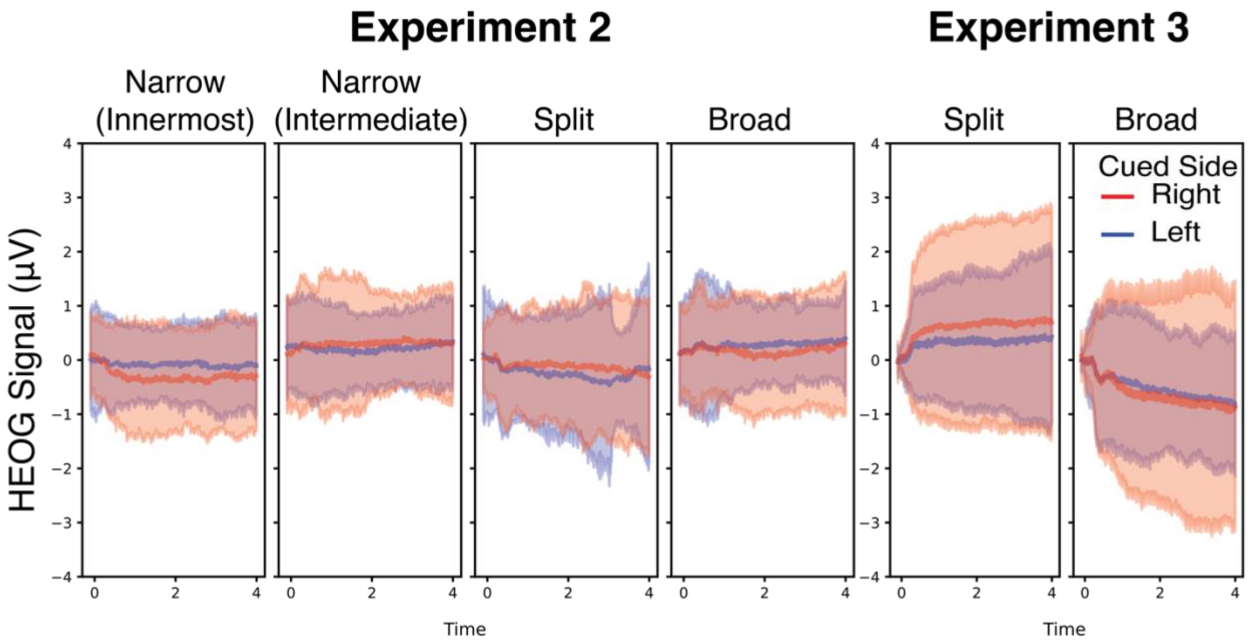

Figure S1. *HEOG activity per focus condition and cue side in Experiments 2 & 3.* The HEOG signal was averaged across trials per focus condition and the cued side for each participant separately. The shaded areas denote for 95% confidence interval after Morey–Cosusineau correction. Each column represent a focus condition. The narrow–focus condition in Experiment 2 contained two configurations in which either the innermost or the intermediate locations were cued, and these configurations were plotted and analyzed separately. Cue side is color coded such that red represents right–side–cued blocks, and the blue represent left–side–cued blocks. The HEOG signal did not vary with focus condition in either experiment. There was a main effect of cue side in both experiments but see the supplementary text for interpretation.

Here, we analyzed the HEOG activity to test whether participants' retinal positions varied across different focus conditions and cue side (left or right visual hemifield). HEOG signals were first baselined to the average signal at the interval from -0.1 s until the flicker onset for each trial. Then, we averaged those trials that belonged to the same cue side and focus condition for each participant. Data from 6 participants in Experiment 2 and 5 participants in Experiment 3 were

discarded due to faulty signal: the HEOG electrodes were detached from skin during the session yielding unusable data. In Figure S1, we show the group average of HEOG activity across time for Experiments 2 and 3 separately for each focus condition and cue side. Positive values indicate eye movements toward the rightwards direction. To test if there was any significant eye movement depending on the focus condition and the cue side, we averaged the HEOG signal across time and ran a two–way repeated–measures ANOVA. In both experiments we found a significant main effect of cue side (see Table S1 & S2). However, there was no main effect of focus condition or an interaction. In Experiment 2, participants' HEOG activity was more positive in the right–side cued trials (0.45 ± 0.14 μV) compared to the left–side cued trials (-0.22 ± 0.14 μV, p_{BH} = 0.009) indicating eye movements towards the cue side. In Experiment 3, participants' HEOG activity was more negative in the right–side cued trials (-0.56 ± 0.14 μV) compared to the left–side cued trials (0.46 ± 0.14 μV, p_{BH} = 0.0001) indicating eye movements towards the opposite side of the cue. Both results suggest that the retinal position of the stimulus did not vary depending on the focus condition in either experiment but might have depended on the cue side. However, we should be cautious of interpreting these effects' significant since the magnitudes of HEOG signal are miniscule and might not correspond to any real eye–movements. According to previous reports a horizontal eye movement of 1-degree visual arc results in ~16 μV activity[1]. Moreover, the direction of the eye movement was towards the cued side in Experiment 2, but it was opposite of the cued side in Experiment 3, which further reduces interpretability of this result.

---

[1] page 199 in Luck, S. J. (2014). *An introduction to the event-related potential technique*. MIT press.

We, also, ran the main ANOVA tests on attention indices in each experiment excluding the participants with faulty HEOG reading (see Tables S3 & S4). The significance and the direction of the factors remained the same as those in the main analysis pipeline.

Table S1. *ANOVA results for average HEOG activity per focus condition and cued side in Experiment 2.*

	F	ε_{GG}	df	P_{BH}	η_p^2	BF	sig.
**Focus**	1.87	0.376	(1.13, 23.67)	0.18	0.08	0.13	
**Side**	11.36		(1, 21)	0.009	0.35	51007510	**
**Focus x Side**	1.98	0.468	(1.4, 29.46)	0.18	0.09	0.13	

Table S2. *ANOVA results for average HEOG activity per focus condition and cued side in Experiment 3.*

	F	ε_{GG}	df	P_{BH}	η_p^2	BF	sig.
**Focus**	0.71		(1,18)	0.41	0.04	0.27	
**Side**	28.12		(1, 18)	10^{-4}	0.61	7992648	***
**Focus x Side**	1.75		(1,18)	0.2	0.09	0.59	

Table S3. *ANOVA results for attention indices per focus condition and cued side in Experiment 2 excluding the participants with faulty HEOG reading.*

	F	ε_{GG}	df	P_{BH}	η_p^2	BF	sig.
**Focus**	11.84	0.705	(1.41, 28.19)	$< 10^{-3}$	0.37	3.74	***
**Location**	0.91		(1, 20)	0.35	0.04	0.68	
**Focus x Location**	1.57	0.950	(1.9, 38.02)	0.22	0.07	0.29	

Table S4. *ANOVA results for attention indices per focus condition and cued side in Experiment 3 excluding the participants with faulty HEOG reading.*

	F	ε_{GG}	df	P_{BH}	η_p^2	BF	sig.
**Focus**	1.03		(1,18)	0.32	0.04	0.34	
**Location**	6.13	0.998	(2, 35.94)	0.005	0.25	44.9	**
**Focus x Location**	0.13	0.862	(1.72, 31.04)	0.85	0.01	0.14	

SNR Topologies per Frequency

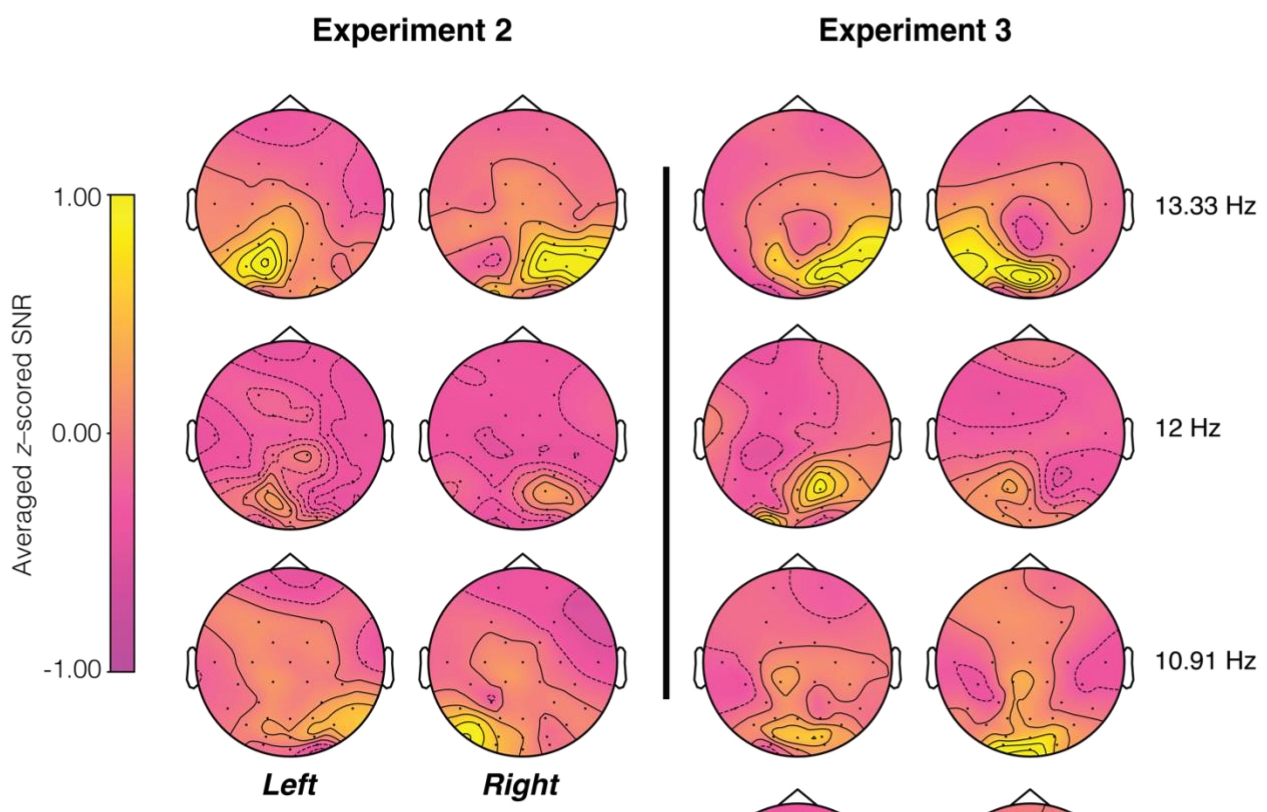

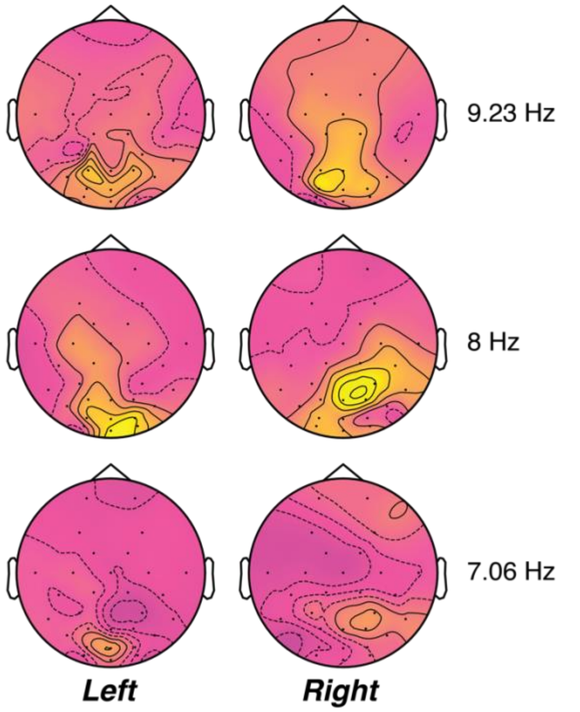

Figure S2. *Average SNR topologies for each stimulation frequency per stimulius side.* SNR topologies are shown for Experiments 2 (left) and 3 (right) for each presentation side and stimulation frequency, separately. Every participants' SNRs at each stimulation side and frequency (averaging across focus conditions) were calculated in the same way as in the main analysis. The resulting SNRs were then z–scored across electrodes after which the grand average is taken across participants and plotted here. Stimulation frequencies induced increased signal in the posterior electrodes in both experiments. No consistent lateralization was observed.
